# Quantifying the basic reproduction number and underestimated fraction of Mpox cases worldwide at the onset of the outbreak

**DOI:** 10.1098/rsif.2023.0637

**Published:** 2024-07-24

**Authors:** Nicola Luigi Bragazzi, Sarafa Adewale Iyaniwura, Qing Han, Woldegebriel Assefa Woldegerima, Jude Dzevela Kong

**Affiliations:** ^1^ Department of Food and Drugs, University of Parma, Parma, Italy; ^2^ Theoretical Biology and Biophysics Group, Los Alamos National Laboratory, Los Alamos, NM, USA; ^3^ Africa-Canada Artificial Intelligence and Data Innovation Consortium (ACADIC), Toronto, Ontario, Canada; ^4^ Department of Mathematics and Statistics, Laboratory for Industrial and Applied Mathematics (LIAM), York University, Toronto, Ontario, Canada; ^5^ Artificial Intelligence and Mathematical Modelling Lab (AIMMLAb), Dalla Lana School of Public Health, University of Toronto, Toronto, Ontario, Canada; ^6^ Global South Artificial Intelligence for Pandemic and Epidemic Preparedness and Response Network (AI4PEP), University of Toronto, Toronto, Ontario M3J 1P3, Canada

**Keywords:** basic reproduction number, Mpox, underestimation, men having sex with men, mathematical modelling, sexually transmitted infections

## Abstract

In 2022, there was a global resurgence of mpox, with different clinical-epidemiological features compared with previous outbreaks. Sexual contact was hypothesized as the primary transmission route, and the community of men having sex with men (MSM) was disproportionately affected. Because of the stigma associated with sexually transmitted infections, the real burden of mpox could be masked. We quantified the basic reproduction number (*R*_0_) and the underestimated fraction of mpox cases in 16 countries, from the onset of the outbreak until early September 2022, using Bayesian inference and a compartmentalized, risk-structured (high-/low-risk populations) and two-route (sexual/non-sexual transmission) mathematical model. Machine learning (ML) was harnessed to identify underestimation determinants. Estimated *R*_0_ ranged between 1.37 (Canada) and 3.68 (Germany). The underestimation rates for the high- and low-risk populations varied between 25–93% and 65–85%, respectively. The estimated total number of mpox cases, relative to the reported cases, is highest in Colombia (3.60) and lowest in Canada (1.08). In the ML analysis, two clusters of countries could be identified, differing in terms of attitudes towards the 2SLGBTQIAP+ community and the importance of religion. Given the substantial mpox underestimation, surveillance should be enhanced, and country-specific campaigns against the stigmatization of MSM should be organized, leveraging community-based interventions.

## 1. Introduction

Case reporting and surveillance (CRS) of real-time data related to infectious diseases are paramount in disease control, even though CRS systems can be subjected to uncertainties that can lead to underestimation, either in terms of under-ascertainment (when not all the cases seek healthcare), under-diagnosis (when cases seek healthcare but are not properly diagnosed), and/or under-reporting or under-notification (when a failure to adequately report and notify diagnosed cases occurs) [1]. Under-ascertainment, under-diagnosis and under-reporting/under-notification may have an enormous impact on the investigation of infectious disease outbreaks and the application of control measures. Thus, they may compromise evidence-based infectious disease prevention and mitigation strategies [2]. Underestimation can also lead to major challenges in analysing the epidemiological characteristics of infectious disease outbreaks and provide inaccurate numerical estimates of the key parameters and variables of interest. Therefore, developing techniques to adjust for under-ascertainment and under-reporting/under-notification has been a central issue for public health, policy- and decision-makers [1,2].

Several communicable disorders are underestimated for various reasons, including stigma-induced under-ascertainment, misdiagnosis, inadequate public health and disease surveillance infrastructure or failure to comply with disease reporting requirements [1]. Infectious diseases can also be underestimated for being mild or asymptomatic or owing to weak/overwhelmed public health infrastructure [1]. Moreover, each country may have different reporting rates, depending on country-specific health system modernization, testing capacity, economic-financial aspects, social characteristics, health literacy, human behaviour dynamics, political factors and country-specific local disease epidemiological trends, among others [3,4].

Without accurate numerical estimates, it is difficult to precisely quantify the proportions of severe and critical cases, which age group is highly affected by the infection and how to allocate resources effectively. Thus, underestimation of the incidence and prevalence of infections can affect the efficiency and reliability of surveillance and notification systems and, thus, the implementation of adequate control measures [4]. Therefore, estimating under-ascertained and under-reported/under-notified cases is crucial for public health interventions. It can improve the accuracy of infectious disease modelling and help inform effective control policies.

The mpox (formerly known as monkeypox) is a viral zoonotic disease caused by an orthopoxviral infectious agent (known as mpox virus, MPV) that results in a smallpox-like disease in humans and some other animals and is recognized as the most critical orthopoxviral infection after the eradication of smallpox [5]. It is endemic in Western and Central Africa, and cases outside of Africa have emerged only in recent years [5,6], including the 2003 outbreak in the United States in which all infections were related to contacts with infected animals. During the 2022 outbreak, mpox disproportionately impacted the community of men who have sex with men (MSM) [5,7], which is a term that refers to a subset of individuals defined by their sexual behaviour rather than their sexual orientation or identity. This term was coined by prominent epidemiologists and is primarily used in public health contexts to address sexual health issues and risks without specifically labelling the sexual orientation of these individuals. The MSM community is incredibly diverse and may include gay and bisexual men (known as gbMSM), as well as men who do not identify as gay or bisexual but who still engage in sexual acts with other men (known as heterosexually identified MSM or hMSM). While there may be overlap, the MSM category is distinct from the broader 2SLGBTQIAP+ community, which includes people of diverse sexual orientations and gender identities, encompassing lesbian, gay, bisexual, transgender, queer or questioning, intersex, asexual, pansexual and Two-Spirit individuals, focusing more on identity rather than specific sexual behaviours.

The first mpox cases were confirmed in early April–May 2022 in the United Kingdom, subsequently spreading to several countries on almost all continents, according to the World Health Organization (WHO) and the US Centers for Disease Control and Prevention (CDC) data. However, it seems that cases were already spreading in Europe much earlier [4,8]. Since then, mpox cases around the globe have increased at a rapid rate. However, at the beginning of the 2022 mpox epidemic, the scale of the outbreak in several countries, including the United States, was bigger than what the CDC and WHO data showed [4,9], probably owing to the ineffectiveness of the testing system (testing was limited and slow), as well as to individuals’ willingness to get tested and report their results.

In this work, we address the issue of underestimation for the 2022 mpox epidemic by applying a novel method to quantify the portion of underestimated cases and the basic reproduction number of the disease in 16 selected countries, since reporting rates for the same infectious disease can vary spatially, with country-specific economy, institutional, social, political and behavioural factors affecting national reporting rates. For this, we formulate a ‘susceptible–exposure–infection–quarantine–recovery’ (SEIQR)-type compartmental model by stratifying the population of each country into high- and low-risk groups based on their sexual behaviour dynamics. We adopted the term high-risk (versus low-risk) referring to the MSM community (versus the general population), to avoid any label or stigma. We identified specific and measurable components of the model for each country-specific estimation rate and then used mpox data of each country to make the estimations. Moreover, we use country-related characteristics, in terms of attitudes towards the MSM community and mpox case definition, to validate our approach and explain differences in underestimating mpox across countries. We anticipate that the approach used in the present work can be leveraged to characterize uncertainty and correct for bias for other infections.

## 2. Methods

### 2.1. Literature review

We searched two major scholarly, electronic databases in the biomedical field (namely, MEDLINE via its openly accessible interface, PubMed and Scopus), from the inception of the 2022 mpox outbreak. To improve the chances of getting all relevant studies that have not been indexed, we also searched Google Scholar. The following keywords were used: ‘monkeypox’, ‘mpox’, ‘monkeypox virus’, ‘mpox virus’, ‘mathematical model’, ‘statistical model’, ‘model-based study’, ‘modeling study’, ‘under-estimation’, ‘under-ascertainment’, ‘under-diagnosis’, ‘under-notification’ and ‘under-reporting’. We included publications written in English, Spanish, Portuguese, French and German. We found that in the existing scholarly literature, only a few efforts have been made to estimate the hidden burden of mpox. For instance, one study [10] proposed a data-driven Chao–Böhning estimator to determine the true number of mpox cases in 10 countries based on the cumulative distributions of weekly reported cases. The authors found that in all the countries studied, there was a relevant proportion of undetected cases, and the estimated ‘true’ number of infections could be more than two to three times the observed cases. In another investigation [11], it was hypothesized that mpox among MSM might be underdiagnosed in European countries with more stigmatizing attitudes towards male homosexuality and limited access to sexually transmitted infection testing for MSM. The author conducted correlation tests between the national incidence of mpox in European countries and the intensity of screening for sexually transmitted infections (STIs) and a composite indicator of 2SLGBTQIAP+ rights known as the Rainbow Index. Positive correlations were found between the national cumulative incidence of mpox and access to chlamydia/gonorrhoea screening, syphilis screening and the Rainbow Index. These correlations reflect broader public health infrastructure and policies: countries with more comprehensive healthcare systems and progressive policies (as indicated by the higher Rainbow Index score) are better at both detecting and reporting mpox cases, and providing access to sexual health services, indicating that where there is better health infrastructure and more inclusive policies, there is also better surveillance and reporting of mpox.

### 2.2. Mathematical model

**Figure 1.**
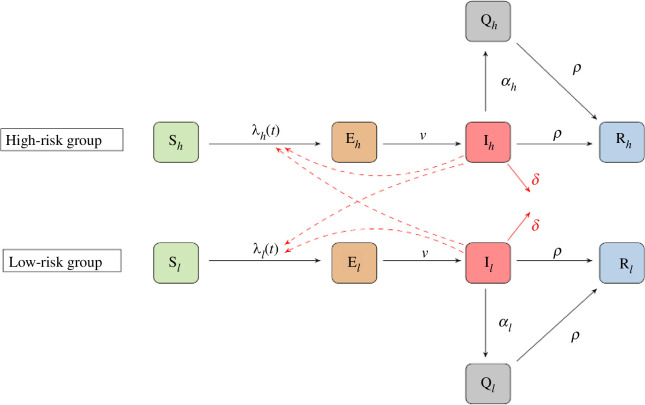
Schematic illustration of the SEIQR model. Model compartments are susceptible (S), exposed (E), infectious (I), quarantined (Q) and recovered/removed (R). The population of each country is divided into high- (subscript *h*) and low-risk (subscript *l*) groups. The black solid arrows indicate the transition of individuals through the different stages of the disease at the rates shown beside the arrows, the red solid arrows indicate mpox-induced death and the red dashed arrows indicate disease transmission.

We use a two-group two-route SEIQR model of mpox [12] to study the early dynamics of the disease in selected countries around the world. The model divides the total population of each country into two groups: high- and low-risk, and considers two routes of mpox transmission: sexual and non-sexual. The population of each group is further divided into five compartments: susceptible (S), exposed (E), infectious (I), quarantined (Q) and recovered/removed (R). Mpox transmission through the sexual route is considered in the high-risk group only since the disease transmission through this route in the low-risk group is minimal [13]. However, the non-sexual transmission route is considered in both groups. An infected individual in either of the two groups can infect susceptible individuals in both groups. As we are interested in studying the early dynamics of mpox, we do not consider the movement of individuals from the high- to the low-risk group and vice versa. In addition, our model does not consider reinfection and natural births or deaths. We assume that infectious individuals quarantine at a constant rate and that quarantine can happen as soon as from the first day of their infectious period. These individuals do not infect others and transition to the recovered compartments upon recovery. A schematic diagram of the model is given in figure 1, where the compartments with subscripts *h* and *l* are for the high- and low-risk groups, respectively. Full differential equations, parameter descriptions and values can be found in the electronic supplementary material.

Using the next-generation matrix approach [14], we derived the basic reproduction number for the high-risk group (*R*_0*h*_) and low-risk group (*R*_0*l*_), and the entire population (*R*_0_; see electronic supplementary material for details).

### 2.3. Data

We collected the main facts about the 2022 mpox multi-country outbreak from major public health authorities [15–17] and daily reported cases (7-day rolling average of confirmed cases) of mpox from Our World in Data [18] for 16 countries, namely, Argentina, Belgium, Brazil, Canada, Chile, Colombia, France, Germany, Italy, Mexico, The Netherlands, Peru, Portugal, Spain, the United Kingdom and the United States. The choice of these countries is determined based on the size of their mpox epidemics and data availability. We consider the early stages of the epidemic (June–September 2022) in each of the countries and assume that there were no control measures in place during this period. Even if this assumption is not completely correct, since some countries, like France, Spain and Italy, had begun implementing public health interventions during the period of our modelling study, so we do not expect this to impact the findings of our simulation.

We aggregate the extracted data into high- and low-risk groups by assuming that all the individuals in the high-risk group are in the MSM community. In addition, since 95% of the reported cases of mpox during the early stages of the epidemics around the world are in the MSM community [7], we extracted 95% of the reported data in each country. We attributed it to the high-risk population and 5% to the low-risk population. Furthermore, we divided the population of each country into high- and low-risk groups based on the implementation of a methodology previously developed [19]. Since currently available data concerning socially marginalized populations are incomplete, lacking or unreliable, and triangulation of different data sources is sometimes unfeasible and technically challenging, the authors devised a passive surveillance-based strategy capturing interest in gay pornographic material displayed on the Internet and social media. In more detail, the Google Trends platform was exploited to approximate the MSM population size estimate by comparing the volume of queries generated by looking for ‘gay porn’ with the volume of searches returned by googling ‘porn’ (see electronic supplementary material, table S2). Of note, when applied to Canada, the MSM population size estimate was in the range of 2–4%, which was twice the estimate provided by conventional techniques [20]. Despite some biases (including variability in the Internet coverage and consumption of gay pornographic material by straight individuals, including women), this method can provide estimates for countries where existing statistics are outdated [21] and/or would fail to meet the WHO and United Nations Programme on HIV/AIDS (UNAIDS) recommendations [22].

The non-sexual contact rates were computed using each country’s demographic age structure and the overall mixing matrix across all locations [23]. The population size, fraction of the high-risk group and non-sexual contact rates for each country are given in electronic supplementary material, table S2.

### 2.4. Bayesian inference

We calibrate our SEIQR model to the daily reported cases (7-day rolling average of confirmed cases) of mpox in each country using a Bayesian inference framework and the RStan package in R version 3.6.3 [24]. For all the results presented in this article, we used the adaptive Hamiltonian Monte Carlo method No-U-Turn sampling (NUTS) in RStan with 5000 iterations and four chains. We estimated the sexual contact rate for the high-risk population, the quarantine rate and the case estimation fractions for the high- and low-risk groups, respectively, from our model calibration for each country. These parameters are used to calculate the basic reproduction number for the high-risk group (*R*_0*h*_), low-risk group (*R*_0*l*_), and the entire population (*R*_0_; see electronic supplementary materials for more details).

### 2.5. Determinants of underestimation

To validate our mathematical model, for each country, we extracted information related to (i) the mpox case definition policy (which specific combination of diagnostic/laboratory, clinical and/or epidemiological criteria was used to define a confirmed, probable, suspected or discarded mpox case), (ii) the mpox case notification policy (which mpox cases—confirmed, probable and/or suspected cases—are notified), (iii) compliance of the competent public health authority with the WHO recommendations [25], (iv) attitudes towards the 2SLGBTQIAP+ community (equality index, legal index and opinion index), and (v) importance of religion, because we hypothesized these parameters could have an impact on 2SLGBTQIAP+ community health-related policies.

More specifically, determinants of underestimation were assessed by hierarchical clustering on mixed principal component analysis (HCmixedPCa). We leveraged a previously published study [25], which showed a substantial degree of heterogeneity and overlap of criteria in mpox case definition in 32 countries. For confirmed cases, only 18 countries (56%) complied with the WHO guidelines and tested for mpox using species-specific polymerase chain reaction (PCR) method and/or sequencing. For probable and suspected cases, seven and eight countries had not released definitions in the publicly available documents/reports, respectively. Of note, none of the countries analysed followed the WHO criteria for probable and suspected cases. For ruled-out cases, definitions were provided only by 13 countries (41%), with only two countries (6%) strictly following and adhering to the WHO recommendations. In terms of mpox case notification, only 12 countries (38%) notified both probable and confirmed cases in accordance with the WHO requirements. Information related to mpox case definition and reporting or notification policies was extracted from [25].

Attitudes towards the 2SLGBTQIAP+ community were extracted from the Equaldex platform (freely accessible) [26]. Equaldex is a collaborative knowledge base for the 2SLGBTQIAP+ community, which systematically collects, displays and analyses data about the 2SLGBTQIAP+ laws, facts, polls and opinions, using a structured, consistent and verified format. The importance of religion by country was extracted from the global 2009 Gallup Poll [27], in which participants were asked, ‘Is religion important in your daily life?‘. Participants could choose among three options: ‘yes’, ‘no’ and ‘don’t know’; or select ‘prefer not to answer’.

## 3. Results

### 3.1. Basic reproduction numbers and underestimation fractions

**Figure 2.**
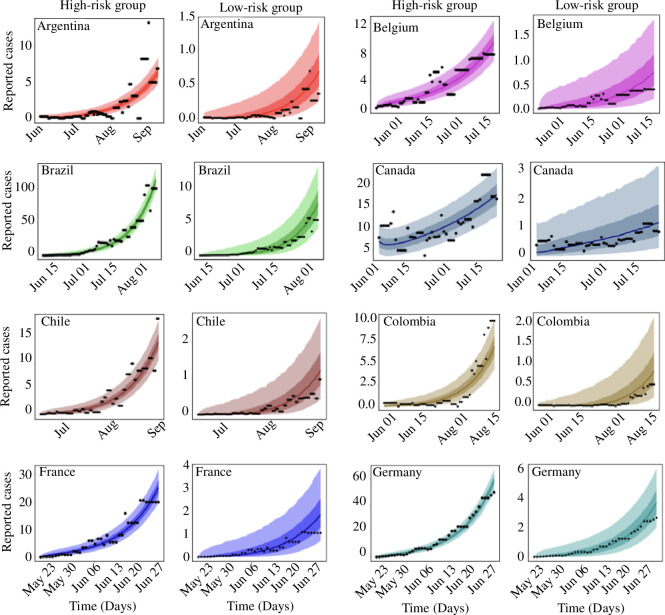
Observed and estimated daily cases of mpox for Argentina, Belgium, Brazil, Canada, Chile, Colombia, France and Germany. Black dots are the daily reported cases of mpox, the solid lines are the median predicted cases, the narrower (darker) bands are the 50% CrI, while the wider (lighter) bands are the 90% CrI.

**Figure 3.**
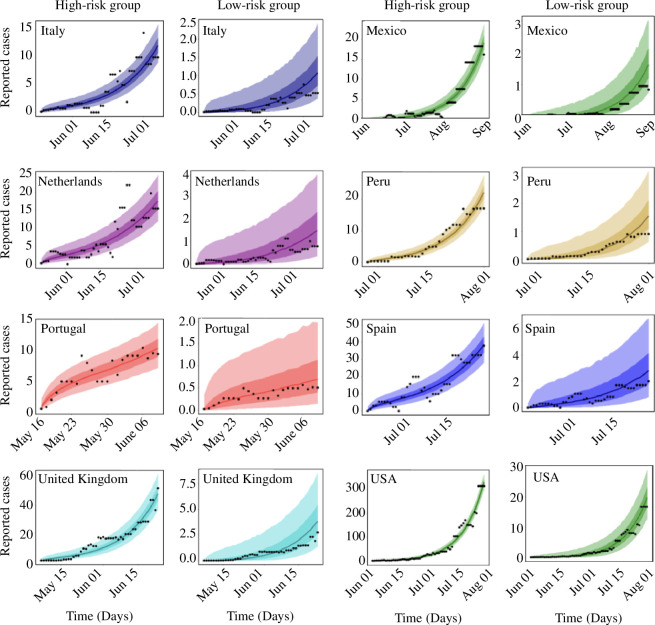
Observed and estimated daily cases of mpox for Italy, Mexico, The Netherlands, Peru, Portugal, Spain, the United Kingdom and the United States. Black dots are the daily reported cases of mpox, the solid lines are the median predicted cases, the narrower (darker) bands are the 50% CrI, while the wider (lighter) bands are the 90% CrI.

The results of our inferences showing the daily reported cases and the predicted cases obtained from our model are presented in figures 2 and 3. The black dots are the daily reported cases (7-day rolling average) of mpox for each country, while the solid lines are the median predicted cases. The narrower (darker) bands are 50% credible intervals (CrI), whereas the wider (lighter) bands are 90% CrI.

**Figure 4.**
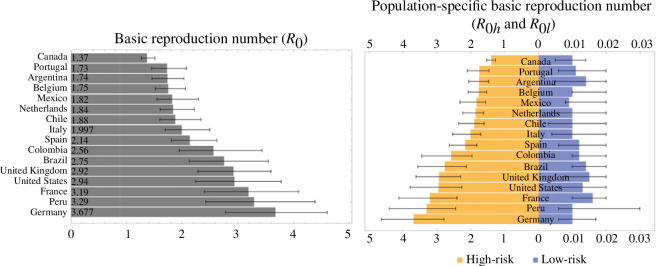
Estimated total basic reproduction number ( $R_{0}$ ) and population-specific basic reproduction numbers ( $R_{0h}$ and $R_{0l}$ ). The estimated basic reproduction number ( $R_{0}$ ) of the mpox epidemic in each country with 90% credible interval (CrI) in increasing order (left) and the population-specific basic reproduction number: high-risk group, $R_{0h}$ (yellow) and low-risk group, $R_{0l}$ (blue) with 90% credible interval (CrI) in increasing order of the former.

The estimated basic reproduction number for the high-risk group (*R*_0*h*_), low-risk group (*R*_0*l*_) and total population (*R*_0_) with 90% CrI are given in figure 4. The estimated basic reproduction numbers range from 1.38 to 3.68 for the high-risk group, 0.009 to 0.016 for the low-risk group, and 1.37 to 3.677 for the total population. The smallest *R*_0_ is obtained for Canada, 1.37 (90% CrI: 1.27–1.51), while the largest is obtained for Germany, 3.677 (90% CrI: 2.78–4.61).

**Figure 5.**
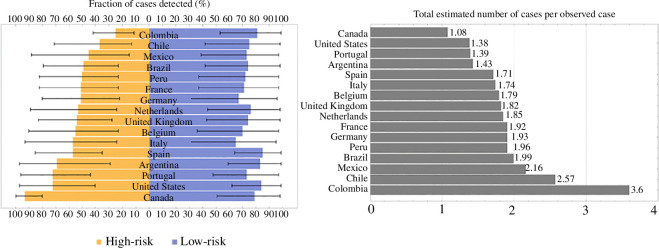
Estimation fractions for high- and low-risk groups and the total number of cases per observed case. The estimated proportion of the total mpox cases detected for the high- and low-risk groups with 90% CrI in increasing order of the former (left), and the total number of estimated cases per observed case in increasing order (right) for each country.

The mean case estimation fractions with 90% CrI for the high- and low-risk groups, respectively, are presented in figure 5. The estimation fraction for the high-risk group ranges from 25% to 93%, where the smallest fraction is obtained for Colombia with a mean estimate of 25% (90% CrI: 11–42%), which implies that an average of only 25% of the total cases of mpox in this country were observed. On the other hand, the highest estimation fraction for the high-risk group was obtained for Canada, with a mean estimate of 93% (90% CrI: 80–99.6%). This corresponds to detecting a mean of 93% of the total daily cases of mpox in the high-risk group in Canada. Similarly, the mean estimation fraction for the low-risk population ranges from 65% to 85%, with Italy having the smallest fraction of 65% (90% CrI: 31–95%) and Spain having the largest fraction of 85% (90% CrI: 64–99%). Combining high- and low-risk case estimation fractions, we obtained the total number of estimated cases for each observed case for each country, shown in figure 5 (right). Overall, Canada has the least under-detected cases, 1.08 behind each observed case, while Colombia has the most unobserved cases, 3.6 behind each observed one.

We also estimated the daily sexual contact rate and the quarantine rate for each country. The estimated daily sexual contact rates are in the range of 1.08–2.05, with the minimum for Argentina (90% CrI: 0.62–2.13), and the maximum for Mexico (90% CrI: 0.83–4.25; see the electronic supplementary material for all the estimated parameters for each country).

### 3.2. Determinants of mpox underestimation

**Figure 6.**
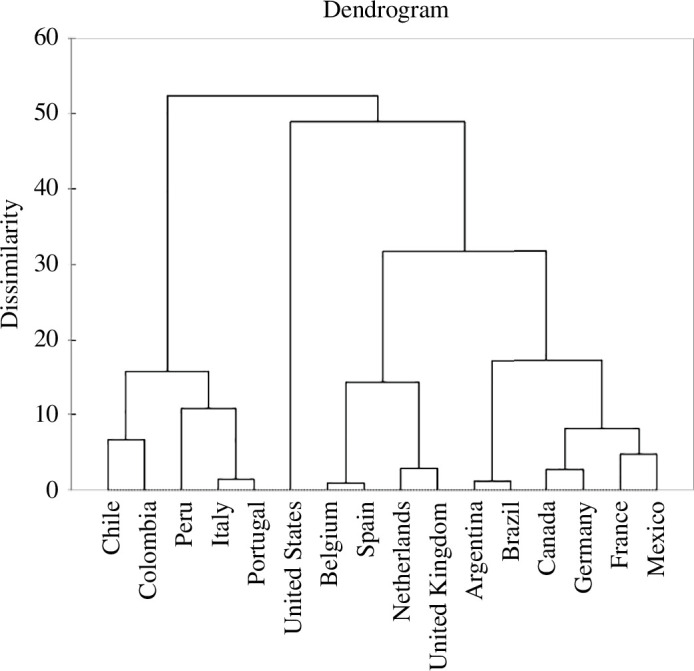
Dendrogram from the hierarchical clustering on mixed principal component analysis (HCmixedPCa) resulting in two clusters. The first cluster includes most of the countries analysed (namely, the USA, Belgium, Spain, The Netherlands, the UK, Argentina, Brazil, Canada, Germany, France and Mexico). The second cluster comprises five countries (Chile, Colombia, Peru, Italy and Portugal).

**Figure 7.**
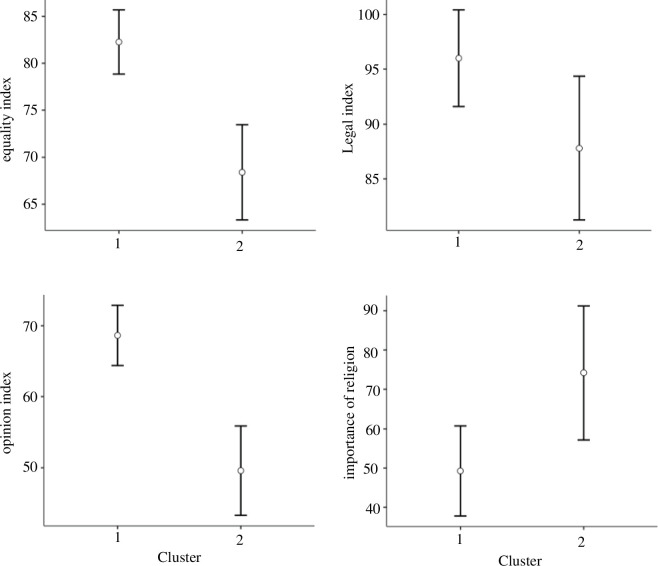
Significant variables characterizing the two clusters. They are attitudes towards the 2SLGBTQIAP+ community (equality, legal and opinion index) and the importance of religion. Cluster 1 includes the USA, Belgium, Spain, The Netherlands, the United Kingdom, Argentina, Brazil, Canada, Germany, France and Mexico. Cluster 2 includes Chile, Colombia, Peru, Italy and Portugal.

HCmixedPCa resulted into two major clusters: Cluster 1 included most of the countries analysed: the United States, Belgium, Spain, The Netherlands, the United Kingdom, Argentina, Brazil, Canada, Germany, France and Mexico, while Cluster 2 comprised Chile, Colombia, Peru, Italy and Portugal (figure 6). The two clusters differed in terms of attitudes towards the 2SLGBTQIAP+ community (equality index, p<0.001 ; legal index, p=0.043 ; and opinion index, p<0.001 ) and the importance of religion ( p=0.021 ), as shown in figure 7.

Of note, the two clusters did not differ in terms of adoption of mpox diagnostic testing criteria (sequencing, p=1.000 ), clinical criteria (mucosal lesions, p=0.299 ; lymphadenopathy, p=0.119 ), epidemiological and additional criteria (epidemiological link, p=1.000 ; self-identification as gay/MSM, p=0.245 ; multiple sexual partners, p=0.588 ; immunoglobulin (Ig) rise, p=1.000 ; orthopoxviral infection, p=1.000 ) for mpox case definition. Similarly, the two clusters did not differ in terms of reporting/notification policies of probable ( p=0.119 ), and suspected mpox cases ( p=0.596 ), as well as compliance with the WHO guidelines ( p=1.000 ). Interestingly, only ‘travel to endemic regions’ was statistically significant ( p=0.036 ), which was a criterion adopted mostly in countries belonging to cluster 2.

## 4. Discussion and limitations

The present study proposes a compartmentalized, risk-structured (high- and low-risk populations), two-route (sexual and non-sexual transmission) mathematical model for capturing the transmission dynamics of mpox. This model enables the computation of the underestimated fraction of mpox cases across several countries. Even though simplified (public health interventions, such as vaccination and herd immunity, are not accounted for), the formulated model can provide clinical public health decision- and policy-makers with useful information that can assist them in designing and implementing effective interventions targeting socially vulnerable communities, such as the 2SLGBTQIAP+ community. It is worth noting that the few analyses available considered the entire population rather than subdividing it into high- and low-risk populations. Moreover, our mathematical model was validated using data specifically focusing on the 2SLGBTQIAP+ community, and we found that attitudes towards this community and the importance of religion were the key factors mostly associated with mpox underestimation rates in a statistically significant fashion.

In this study, we calibrated the SEIQR model of mpox [12] with a risk-structured population, i.e. high-risk and low-risk groups, and two transmission routes, i.e. sexual and non-sexual transmission, reflecting the epidemiology of the 2022 outbreak and in line with studies [28,29] that used transmission models calibrated using real-world data on sexual partnerships. These models revealed that the distribution of sexual partners within the MSM community is heavily skewed, with a small number of individuals having a disproportionately large number of partners, which can explain our relatively high estimated daily sexual contact rates of 1.08–2.05. This kind of distribution can, indeed, drive the continuous increase in mpox cases within this group, even if such patterns were not evident in previous outbreaks. Furthermore, we estimated the basic reproduction numbers and estimation proportions of mpox in 16 countries, selected based on their mpox epidemic size and data availability, by using a Bayesian inference framework.

We formulated the compartmentalized model by assuming that the high-risk group consists of the MSM population only and determined the fraction of the population of each country in the high-risk groups using a passive surveillance-based strategy leveraging Google Trends [19]. Based on the reports available, almost 95% of mpox cases around the world during the early stages of the outbreak are in the MSM community [5,7], and thus, we consider that 95% of the total cases of mpox in each country are in the high-risk group, while the remaining 5% are in the low-risk groups. The daily reported cases data were based on a 7-day rolling average of confirmed cases of mpox in the countries and were obtained from Our World in Data [18]. In addition to the basic reproduction number, we estimated the percentage of the total cases of mpox observed in the high- and low-risk groups for each country.

In the extant scholarly literature, there are a few attempts to estimate the ‘hidden’ burden of mpox, which appears to be quite consistent [30,31], well extending beyond asymptomatic cases, which, according to a recently published meta-analysis [32], occur in 10.2% (95% CI 2.5−17.9%) of instances. For instance, Böhning *et al*. have proposed a lower-bound, data-driven Chao–Böhning estimator for the true number of mpox cases, computed from the cumulative distributions of weekly cases reported in 10 countries [10]. The authors found that the proportion of undetected cases was relevant in all the countries under study, with countries whose estimated ‘true’ number of infections could be more than two to three times the observed one. In more detail, the median value of the ratio between the total estimated and observed cases was 2.7. France had the highest median value (3.16), followed by Brazil (3.14) and the United Kingdom (3.10). Portugal had the lowest (1.82). In our study, the median value of the ratio is 1.8, lower than the value computed by Böhning *et al*. [10]; Colombia has the highest ratio (3.60), followed by Chile (2.57) and Mexico (2.16) and Canada has the lowest value (1.08).

In another investigation, it was hypothesized that mpox among MSM might be underdiagnosed in European countries with more stigmatizing attitudes towards homosexuality and less access to STI testing for MSM [11]. The author correlated the national incidence of mpox in European countries with the intensity of screening for STIs and a composite indicator of 2SLGBTQIAP+ rights—namely, the Rainbow Index, by using a non-parametric test (the Spearman’s rho correlation test). The author found a positive correlation between the national cumulative incidence of mpox and access to chlamydia/gonorrhoea ( ρ of 0.68, *p* < 0.0001), and syphilis screening ( ρ of 0.62, *p* < 0.0001). A positive correlation was also computed with the Rainbow Index ( ρ of 0.65, *p* < 0.0001). On the other hand, the analysis was carried out using the whole population instead of limiting it to the MSM community.

Furthermore, we were able to validate our mathematical model by means of the analysis of the determinants of mpox underestimation. We found that attitudes towards the 2SLGBTQIAP+ community and the importance of religion were the key factors most associated with mpox underestimation rates, regardless of differences in mpox case definition and the type of surveillance system implemented. This has major practical implications, in that, considering the significant underestimation of mpox, it is imperative to take proactive measures to address the issue of health disparities and gaps in the 2SLGBTQIAP+ community. As such, adopting a multi-pronged approach can play a crucial role. Besides harmonizing mpox case definition across the countries and improving the surveillance systems, organizing campaigns against the stigmatization of MSM is paramount. Developing appropriate mpox-related stigma reduction communication strategies and initiatives would increase access to testing facilities, promote routine testing and treatment and enhance data collection methods by using, for instance, techniques of ‘participatory queer epidemiology’, leading to a more accurate depiction of the actual burden of mpox. The active involvement and engagement of 2SLGBTQIAP+ community ambassadors could effectively help health authorities devise and implement targeted and effective strategies to raise awareness, promote inclusivity, foster a more accepting and supportive environment and reduce the negative attitudes surrounding MSM, combating the spread of mpox. Community-based interventions have shown, indeed, promise in reaching vulnerable populations, including MSM, promoting health-seeking behaviours and identifying and removing any barriers that may hinder access to healthcare facilities. Moreover, it is essential to recognize that different countries have unique features in terms of socio-economic and clinico-epidemiological public health characteristics, cultural norms and healthcare systems, which may influence how mpox cases are reported and managed and may contribute to varying degrees of under-reporting in mpox cases. Consequently, when conducting studies to evaluate the effectiveness of community-based interventions, these contextual factors must be carefully considered.

The present study suffers from a number of limitations that should be acknowledged, including the estimation of under-reporting in the initial spread of mpox, which can be a complex, challenging task that involves a range of epidemiological, mathematical and statistical methods, with the accuracy of these estimates being significantly influenced by the assumptions made in the analysis. It should be emphasized that the quality and availability of data play a critical role, which limited our analysis to a set of 16 countries. Initial spread data can come from various sources, including healthcare facilities, laboratories and public health surveillance systems, and the completeness and accuracy of this data are crucial for reliable estimates. On the other hand, the potential impact of these variables was statistically tested, even if another shortcoming is represented by the limited list of parameters chosen for the assessment of the determinants of mpox underestimation, which is far from exhaustive. Therefore, future studies should explore a wider array of socio-economic and clinical public health characteristics at the country level in a more systematic fashion. Background immunity, conferred by compulsory smallpox vaccination, was not accounted for in our modelling, since its effects, if any, would have been marginal, in that they are limited to the elderly population, while the sexually active population involved in mpox transmission dynamics is significantly younger. Finally, we assumed that no public health measures were implemented, and no herd immunity was still achieved during the entire study period. Even if this may be a relatively accurate approximation of the reality—the roll-out and uptake of mpox vaccine were extremely limited during the time period under study, as well as the effects of herd immunity—this may have resulted in a slight overestimation of the underestimated fraction of mpox cases. Further research is warranted to investigate the impact of mpox vaccination and herd immunity on mpox underestimation. Immuno-epidemiological surveys are anticipated to play a key role in this regard. Preliminary findings show that in the United States and in other areas in North America, the spread of mpox started to decrease before vaccine-induced immunity had reached more than 10% of high-risk individuals [31].

On the other hand, even though simplified, the proposed model can provide clinical public health decision- and policymakers with useful information that can assist them in designing and implementing effective interventions targeting socially vulnerable communities, such as the 2SLGBTQIAP+ community.

In conclusion, factors such as stigma, cultural beliefs, accessibility of healthcare services and legal frameworks can impact the willingness of individuals to disclose their mpox status or seek medical attention. Understanding these differences is vital in developing tailored and multi-faceted interventions that can effectively address under-reporting and ultimately lead to more impactful and sustainable clinical public health outcomes.

Our model has the potential to offer valuable insights to clinical public health decision-makers and policymakers. It can aid them in creating and executing effective interventions specifically focused on socially vulnerable communities, like the 2SLGBTQIAP+ community. Our research stressed the roles of aspects like stigma, cultural beliefs, healthcare accessibility and legal frameworks that can influence people’s inclination to disclose their mpox status or seek medical help. Recognizing these factors is crucial for developing customized and comprehensive packages of interventions that can effectively tackle under-reporting and result in more significant and lasting improvements in clinical public health.

## Data Availability

The mpox case data used for our study is publicly available and can be obtained from Our World in Data [33]. Supplementary material is available online [34].

## References

[1] Gibbons CL *et al* . 2014 Measuring underreporting and under-ascertainment in infectious disease datasets: a comparison of methods. BMC Public Health **14** , 1–7. (10.1186/1471-2458-14-147) 24517715 PMC4015559

[2] Meadows AJ *et al* . 2022 Infectious disease underreporting is predicted by country-level preparedness, politics, and pathogen severity. Health Secur. **20** , 331–338. (10.1089/hs.2021.0197) 35925788 PMC10818036

[3] Albani V , Loria J , Massad E , Zubelli J . 2021 COVID-19 Underreporting and its impact on vaccination strategies. BMC Infect. Dis. **21** , 1111. (10.1186/s12879-021-06780-7) 34711190 PMC8552982

[4] Bragazzi NL *et al* . 2022 Knowing the unknown: the underestimation of monkeypox cases. insights and implications from an integrative review of the literature. Front. Microbiol. **13** , 1011049. (10.3389/fmicb.2022.1011049) 36246252 PMC9563713

[5] Bragazzi NL , Kong JD , Mahroum N , Tsigalou C , Khamisy-Farah R , Converti M , Wu J . 2023 Epidemiological trends and clinical features of the ongoing monkeypox epidemic: a preliminary pooled data analysis and literature review. J. Med. Virol. **95** , e27931. (10.1002/jmv.27931) 35692117

[6] Kumar N , Acharya A , Gendelman HE , Byrareddy SN . 2022 The 2022 outbreak and the pathobiology of the monkeypox virus. J. Autoimmun. **131** , 102855. (10.1016/j.jaut.2022.102855) 35760647 PMC9534147

[7] Thornhill JP *et al* . 2022 Monkeypox virus infection in humans across 16 countries—April–June 2022. N. Engl. J. Med. **387** , 679–691. (10.1056/NEJMoa2207323) 35866746

[8] Guarner J , Del Rio C , Malani PN . 2022 Monkeypox in 2022—what clinicians need to know. JAMA **328** , 139–140. (10.1001/jama.2022.10802) 35696257

[9] Nuzzo JB , Borio LL , Gostin LO . 2022 The WHO declaration of monkeypox as a global public health emergency. JAMA **328** , 615–617. (10.1001/jama.2022.12513) 35895041

[10] Böhning D , Rocchetti I , Maruotti A , Holling H . 2020 Estimating the undetected infections in the COVID-19 outbreak by harnessing capture–recapture methods. Int. J. Infect. Dis. **97** , 197–201. (10.1016/j.ijid.2020.06.009) 32534143 PMC7286831

[11] Kenyon C . 2022 Is monkeypox being underdiagnosed in countries with more stigmatizing attitudes towards men who have sex with men? A simple ecological analysis. Epidemiologia (Basel) **3** , 363–368. (10.3390/epidemiologia3030028) 36417244 PMC9620899

[12] Bragazzi NL , Han Q , Iyaniwura SA , Omame A , Shausan A , Wang X , Woldegerima WA , Wu J , Kong JD . 2023 Adaptive changes in sexual behavior in the high-risk population in response to human monkeypox transmission in Canada can control the outbreak: insights from a two-group, two-route epidemic model. J. Med. Virol. **95** , e28575. (10.1002/jmv.28575) 36772860

[13] Jezek Z , Grab B , Szczeniowski MV , Paluku KM , Mutombo M . 1988 Human monkeypox: secondary attack rates. Bull. World Health Organ. **66** , 465–470. 2844429 PMC2491159

[14] van den Driessche P , Watmough J . 2002 Reproduction numbers and sub-threshold endemic equilibria for compartmental models of disease transmission. Math. Biosci. **180** , 29–48. (10.1016/s0025-5564(02)00108-6) 12387915

[15] World Health Organization . Monkeypox: key facts. See https://www. who.int/news-room/fact-sheets/detail/monkeypox (accessed 26 August 2022).

[16] Centers for Disease Control and Prevention . Impact of monkeypox outbreak on select behaviors. See https://archive.cdc.gov/www_cdc_gov/poxvirus/mpox/response/2022/amis-select-behaviors.html.

[17] Government of Canada . Monkeypox (orthopoxvirus simian). See https://www.canada.ca/en/public-health/services/diseases/monkeypox.html (accessed 26 August 2022).

[18] Mathieu E , Spooner F , Dattani S , Ritchie H , Roser M . 2022 Monkeypox. Our World in Data. See https://ourworldindata.org/monkeypox.

[19] Card KG , Lachowsky NJ , Hogg RS . 2021 Using Google Trends to inform the population size estimation and spatial distribution of gay, bisexual, and other men who have sex with men: proof-of-concept study. JMIR Public Health Surveill. **7** , e27385. (10.2196/27385) 34618679 PMC8669582

[20] Statistics Canada . A statistical portrait of Canada’s diverse LGBTQ2+ communities. See https://www150.statcan.gc.ca/n1/daily-quotidien/210615/dq210615a-eng.htm (accessed 24 August 2022).

[21] Marcus U , Hickson F , Weatherburn P , Schmidt AJ , Network E . 2013 Estimating the size of the MSM populations for 38 European countries by calculating the survey-surveillance discrepancies (SSD) between self-reported new HIV diagnoses from the European MSM Internet survey (EMIS) and surveillance-reported HIV diagnoses among MSM in 2009. BMC Public Health **13** , 919. (10.1186/1471-2458-13-919) 24088198 PMC3850943

[22] World Health Organization (WHO) and the joint United Nations Programme on HIV/AIDS (UNAIDS) . 2020 Key populations strategic information: recommended population size estimates of men who have sex with men. See https://www.unaids.org/sites/default/files/media_asset/2020-recommended-population-size-estimates-of-men-who-have-sex-with-men_en.pdf.

[23] Prem K , van Zandvoort K , Klepac P , Eggo RM , Davies NG , Cook AR , Jit M . 2021 Projecting contact matrices in 177 geographical regions: an update and comparison with empirical data for the COVID-19 era. PLoS Comput. Biol. **17** , e1009098. (10.1371/journal.pcbi.1009098) 34310590 PMC8354454

[24] Stan Development Team . 2020 RStan: the R interface to stan. R package version 2.21.2. See https://mc-stan.org/rstan/.

[25] Panag DS *et al* . 2023 Variations in national surveillance reporting for mpox virus: a comparative analysis in 32 countries. Front. Public Health. **11** , 1178654. (10.3389/fpubh.2023.1178654) 37143972 PMC10151817

[26] Equaldex . LGBT rights by country & travel guide. See https://www.equaldex.com.

[27] Crabtree S . 2010 Religiosity highest in world’s poorest nations. United States is among the rich countries that buck the trend. See https://news.gallup.com/poll/142727/Religiosity-Highest-World-Poorest-Nations.aspx (accessed 31 August 2010).

[28] Endo A , Murayama H , Abbott S , Ratnayake R , Pearson CAB , Edmunds WJ , Fearon E , Funk S . 2022 Heavy-tailed sexual contact networks and monkeypox epidemiology in the global outbreak, 2022. Science **378** , 90–94. (10.1126/science.add4507) 36137054

[29] Brand SPC *et al* . 2023 The role of vaccination and public awareness in forecasts of mpox incidence in the United Kingdom. Nat. Commun. **14** , 4100. (10.1038/s41467-023-38816-8) 37433797 PMC10336136

[30] Miura F , Backer JA , van Rijckevorsel G , Bavalia R , Raven S , Petrignani M , Ainslie KEC , Wallinga J . 2024 Time scales of human mpox transmission in The Netherlands. J. Infect. Dis. **229** , 800–804. (10.1093/infdis/jiad091) 37014716 PMC10938196

[31] Paredes MI , Ahmed N , Figgins M , Colizza V , Lemey P , McCrone JT , Müller N , Tran-Kiem C , Bedford T . 2023 Early underdetected dissemination across countries followed by extensive local transmission propelled the 2022 mpox epidemic. medRxiv 2023. (10.1101/2023.07.27.23293266)

[32] Satapathy P *et al* . 2022 Potentially asymptomatic infection of monkeypox virus: a systematic review and meta-analysis. Vaccines (Basel) **10** , 2083. (10.3390/vaccines10122083) 36560493 PMC9784491

[33] Mathieu E , Spooner F , Dattani S , Ritchie H , Roser M . 2022 Mpox. See https://ourworldindata.org/mpox.

[34] Bragazzi N , Iyaniwura SA , Han Q , Woldegerima WA , Kong JD . 2024 Supplementary material from: Quantifying the basic reproduction number and underestimated fraction of Mpox cases worldwide at the onset of the outbreak. Figshare. (10.6084/m9.figshare.c.7355000) PMC11267235 39044633

